# Digestive enzymes of fungal origin as a relevant cause of false positive *Aspergillus* antigen testing in intensive care unit patients

**DOI:** 10.1007/s15010-020-01506-4

**Published:** 2020-09-03

**Authors:** Ines Schroeder, Karl Dichtl, Uwe Liebchen, Johannes Wagener, Michael Irlbeck, Michael Zoller, Christina Scharf

**Affiliations:** 1 Department of Anesthesiology, University Hospital, LMU München, Marchioninistrasse 15, 81377 Munich, Germany; 2 Max Von Pettenkofer-Institut für Hygiene Und Medizinische Mikrobiologie, LMU München, Medizinische Fakultät, Munich, Germany; 3 Institut für Hygiene Und Mikrobiologie, Julius-Maximilians-Universität Würzburg, Würzburg, Germany; 4 National Reference Center for Invasive Fungal Infections (NRZMyk), Jena, Germany

**Keywords:** Invasive aspergillosis, Critical illness, Galactomannan antigen assay, False positive results, Nortase, Digestive enzymes of fungal origin

## Abstract

**Background:**

Galactomannan antigen (GM) testing is widely used in the diagnosis of invasive aspergillosis (IA). Digestive enzymes play an important role in enzyme substitution therapy in exocrine pancreatic insufficiency. As digestive enzymes of fungal origin like Nortase contain enzymes from *Aspergillus*, a false-positive result of the test might be possible because of cross-reacting antigens of the cell wall of the producing fungi. We, therefore, asked whether the administration of fungal enzymes is a relevant cause of false-positive GM antigen test results.

**Methods:**

Patients with a positive GM antigen test between January 2016 and April 2020 were included in the evaluation and divided into two groups: group 1—Nortase-therapy, group 2—no Nortase-therapy. In addition, dissolved Nortase samples were analyzed in vitro for GM and β-1,3-D-glucan. For statistical analysis, the chi-squared and Mann‒Whitney *U* tests were used.

**Results:**

Sixty-five patients were included in this evaluation (30 patients receiving Nortase and 35 patients not receiving Nortase). The overall false positivity rate of GM testing was 43.1%. Notably, false-positive results were detected significantly more often in the Nortase group (73.3%) than in the control group (17.1%, *p* < 0.001). While the positive predictive value of GM testing was 0.83 in the control group, there was a dramatic decline to 0.27 in the Nortase group. In vitro analysis proved that the Nortase enzyme preparation was highly positive for the fungal antigens GM and β-1,3-D-glucan.

**Conclusions:**

Our data demonstrate that the administration of digestive enzymes of fungal origin like Nortase leads to a significantly higher rate of false-positive GM test results compared to that in patients without digestive enzyme treatment.

## Introduction

Invasive aspergillosis (IA) is a significant cause of morbidity and mortality in immunocompromised patients [1]. Diagnosis of IA remains challenging. In 2019, the consensus committee of the European Organization for Treatment of Cancer/Mycoses and the Mycoses Study Group (EORTC/MSG) proposed their revised definition for the diagnosis of invasive fungal infections. IA is diagnosed based on findings in imaging studies, host factors, histopathology, and microbiology [2]. Since the sensitivity of *Aspergillus* culture or microscopy in blood and bronchoalveolar lavage (BAL) is often poor, *Aspergillus* antigen testing is additionally recommended for patients who are at risk or suspicion of IA [3–5].

Galactomannan (GM) is a polysaccharide and a major constituent of cell walls of *Aspergillus* species. It can be detected in the host’s bloodstream during invasive infection [6]. The GM test is a double-sandwich enzyme-linked immunosorbent assay (ELISA), which is widely used for testing serum and BAL fluid for IA. Sensitivity of GM testing for IA ranges from 70 to 85%, and specificity from 78 to 91%, in immunocompromised patients [7]. Insufficient data are available regarding the test’s performance in patients following solid organ transplantation. Sensitivity and specificity might be decreased in this setting, presumably due to less angioinvasive growth in patients with better immune defense compared to neutropenic patients [8, 9].

Digestive enzyme preparations are used for enzyme substitution therapy in exocrine pancreatic insufficiency [10]. The advantage of Nortase, a digestive enzyme of fungal origin (DEFO) in the ICU setting, in contrast to alternative formulas is that the capsule can be opened, and the contained powder is stable to gastric acidity. Therefore, enteral application through a feeding tube is feasible. Hence, it is the preferred substance used in enteral nutrition of patients with exocrine pancreas insufficiency, who are unable to take medication orally—in our setting, critically ill patients following major surgery. The powder contains fungal enzymes synthesized by *Aspergillus oryzae* that contains also GM in their cell wall like *Aspergillus fumigatus*. Enzymes derived from microbial sources can be obtained over the counter and have a long history of safe use within the food industry.

A high number of GM antigenemia episodes was noticed in patients receiving Nortase on the Intensive Care Units (ICU) of a University hospital. False positive results have not been associated with the administration of digestive enzymes before. The aim of this study was to investigate whether patients receiving Nortase have a higher rate of false-positive test results in GM testing.

## Methods

### Study setting

This is a monocentric, retrospective observational study investigating the influence of the application of Nortase on the performance of the GM ELISA. A local institutional review board approved the study (registration number 20-269).

### Laboratory testing

GM measurement was performed using the Platelia *Aspergillus* antigen ELISA (Bio-Rad Laboratories, Hercules, CA, USA). Optical density (OD) indices were rounded to one decimal place, with a lower limit of 0.1. A GM OD index ≥ 0.5 in two sequential samples including the retesting of the first positive sample was considered positive. β-1,3-D-glucan (BDG) analysis was conducted using the Wako BDG assay (FUJIFILM Wako Chemicals Europe, Neuss, Germany).

We typically use Nortase (Repha, Langenhagen) as a digestive enzyme preparation in the clinical setting. Detailed product information, especially regarding the composition of the capsule, can be found in the summary of product characteristics.

In vitro analysis was performed on five Nortase samples. Nortase capsules were opened and suspended in 10 mL of normal saline each. The solution was agitated and subsequently processed like the serum samples. Three different batches were used to exclude contamination by batch.

### Study population

All GM-seropositive patients treated at two ICUs of the university hospital of Munich between January 2016 and April 2020 were included in the evaluation. Subsequently, they were divided into two groups: group 1, receiving Nortase; and group 2, not receiving Nortase. Five to 15 capsules Nortase were administered two to four times daily determined by the attending physician. GM testing was performed twice a week, within the clinical routine of our ICUs. In addition, all patients receiving Nortase between January 2016 and April 2020 during their stay at the ICUs were evaluated for GM antigenemia.

### Data collection

The charts of all patients were reviewed for the diagnosis of IA according to the criteria of the EORTC/MSG consensus group [2]. However, GM testing is a major factor in these criteria. In consideration of the question addressed by this study, the more restrictive categories “probable* IA” and “possible* IA” had to be applied, which are characterized by excluding GM from the criteria for categorization (Table 1). To strictly identify those patients who had no evidence of IA other than GM antigenemia with respect to the clinical characteristics of the study collective (ICU patients rather than hematology‒oncology patients), an additional category was applied, i.e., “presumable IA”. All patients who did not fulfill the EORTC/MSG criteria for IA but showed evidence for IA in two EORTC criteria (clinical features, mycological evidence other than GM, host factors) were declared as presumable. Accordingly, GM false positivity was defined as positive GM test results in patients without any further evidence of IA.

**Table 1:** EORTC/MSG and modified definitions of probability of invasive aspergillosis

Category	Host factor	Clinical feature	Mycological evidence
Probable IA	+	+	+
Probable* IA	+	+	+ *
Possible IA	+	+	–
Possible* IA	+	+	–*
*Presumable IA#*	±	±	+ */–
Proven IA		Microscopic or cultural finding of *Aspergillus* spp. from sterile material	
*No evidence for IA*		≤ 1 criterion of probable IA*	

Note: Modified categories in italics; *IA* invasive aspergillosis; +* evidence other than GM seropositivity; –* no evidence apart from GM seropositivityote; ^#^for category presumable 2 criteria must be fulfilled

### Statistical analysis

Statistical analysis was performed with IBM SPSS statistics (version 26.0, IBM Corp., Armonk, NY, USA). The dataset was evaluated with a focus on significant changes or differences in the rate of false-positive test results in both the groups. Therefore, the chi-squared test and the Mann‒Whitney *U* test were used.

## Results

### GM antigenemia in DEFO patients

Between January 2016 and April 2020, 40 patients at our ICUs received Nortase. Thirty (75%) of them developed GM antigenemia and were included in the study.

### Demographic and clinical data of GM positive individuals

In the study period, a total of 65 patients at our ICUs tested positive for GM. Thirty patients receiving Nortase were allocated to group 1, and 35 others were placed in group 2. As all patients were critically ill, immunocompromised, and often had undergone recent surgery, intense and invasive diagnostic procedures (for example, lung biopsies) were performed to rule out IA. For detailed patient characteristics and diagnostic workup, see Table 2.

**Table 2:** Patient characteristics and diagnostic work up in both groups

	Group1 “Nortase therapy”: *n* (%) or median [range]	Group2 “no Nortase therapy”: *n* (%) or median [range]
Number of patients	30 (46.2%)	35 (53.8%)
Age: years	37 [21, 67]	58 [22, 79]
Gender: male/female	13/17 (43.3%/56.7%)	19/16 (54.3%/45.7%)
Cystic fibrosis	27 (90%)	2 (5.7%)
Solid organ transplantation	28 (93.3%)	19 (54.3%)
Sepsis	0 (0%)	7 (17.1%)
ARDS	0 (0%)	6 (11.4%)
Microbiological workup (i.e., culture from BAL or sputum, urine, blood, sterile body fluid samples)	30 (100%)	35 (100%)
Cultivation of *Aspergillus* spp.	8 (26.7%)	26 (74.3%)
CT of body site suspicious for IA	29 (96.7%)	35 (100%)
CT positive for IA*	4 (13.3%)	17 (48.6%)
Histopathology of biopsy from body site suspicious for IA	28 (93.3%)	24 (68.6%)
Histopathology positive for invasive mold infection	0 (0%)	10 (28.6%)
Performed clinical examination	30 (100%)	35 (100%)
In-hospital mortality	17 (56.7%)	21 (60%)
Autopsy performed	2 (7%)	3 (8.6%)
Autopsy positive for IA	0 (0%)	2 (5.7%)
Antifungal therapy of IA	24 (80%)	31 (88.6%)

Note: *BAL* bronchoalveolar lavage, *IA* invasive aspergillosis, *ARDS* Acute respiratory distress syndrome, *CT* Computed tomography

*According to the criteria of the EORTC/MSG consensus guidelines [2]

The most frequent reason for the application of Nortase was the necessity of enteral feeding through an enteral feeding tube of patients with exocrine pancreatic insufficiency due to underlying cystic fibrosis following lung transplantation (27/30 patients). Three patients had different reasons for exocrine pancreatic insufficiency.

### Assessment of validity of GM testing

**Fig. 1:**
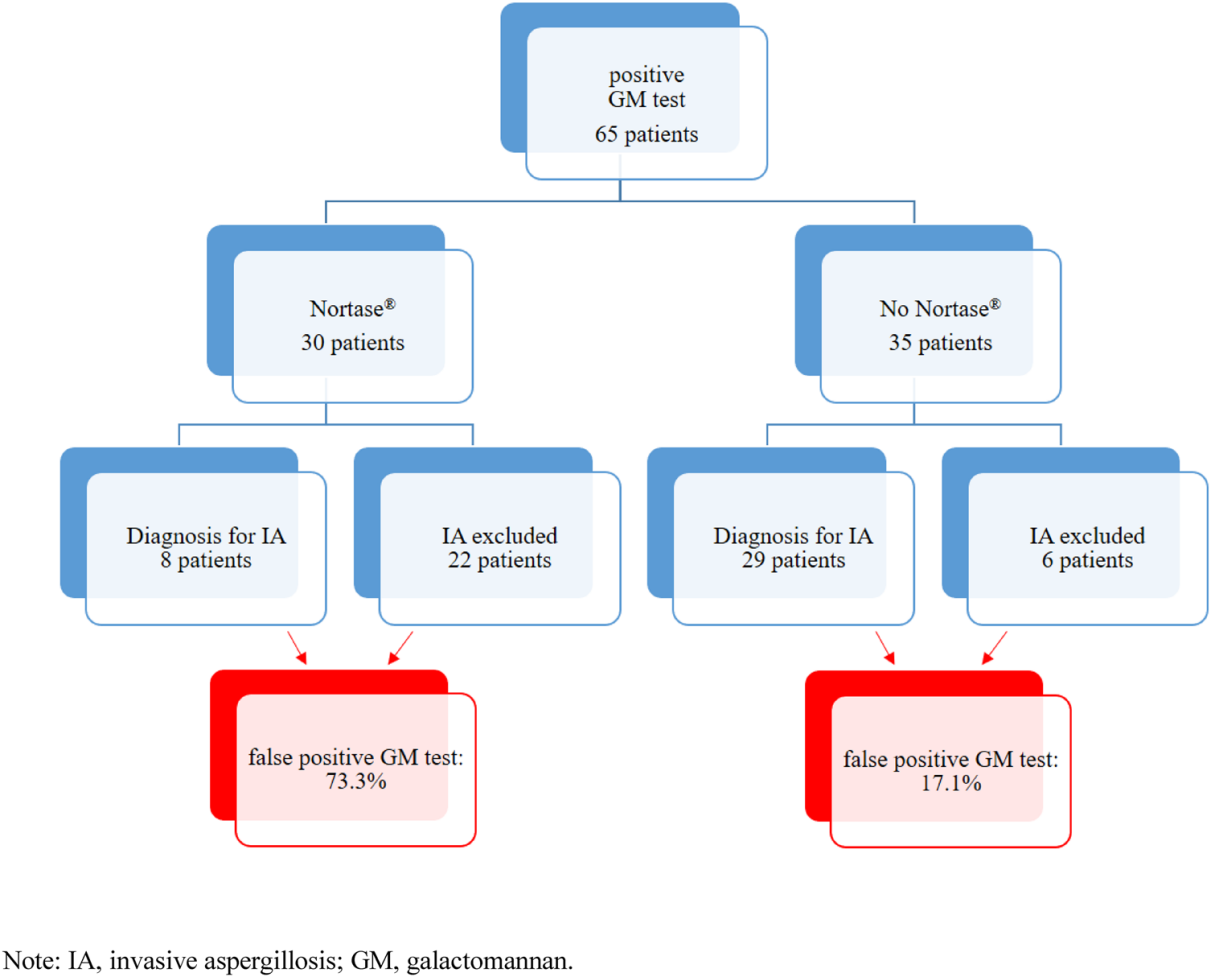
Distribution of false-positive GM test results in both groups

In the Nortase group, patients were diagnosed with proven, probable*, possible* and presumable IA according to the (modified) EORTC/MSG criteria in 0, 4, 0 and 4 cases respectively (Fig. 1) [2]. Twenty-two cases positive for GM did not meet any of the clinical, histopathological or microbiological criteria, apart from antigenemia, allowing the diagnosis of proven, probable*, possible* or presumable IA. All of these cases underwent an intensive diagnostic workup, including CT scan and histopathology of the suspicious body site. Autopsies were performed on two deceased patients and showed no sign of IA. Consequently, these 22 patients (73.3%) with GM antigenemia as the only parameter indicating IA were considered as false-positive.

In contrast, 8, 11, 1 and 9 patients allocated to group 2 were diagnosed with proven, probable*, possible*, and presumable IA, respectively, according to the (modified) EORTC/MSG criteria [2]. The remaining six patients (17.1%) with GM antigenemia as the only parameter indicating IA were considered false positives (see Fig. 1). For details on patient characteristics classified as presumable IA, see Table 3.

**Table 3:**
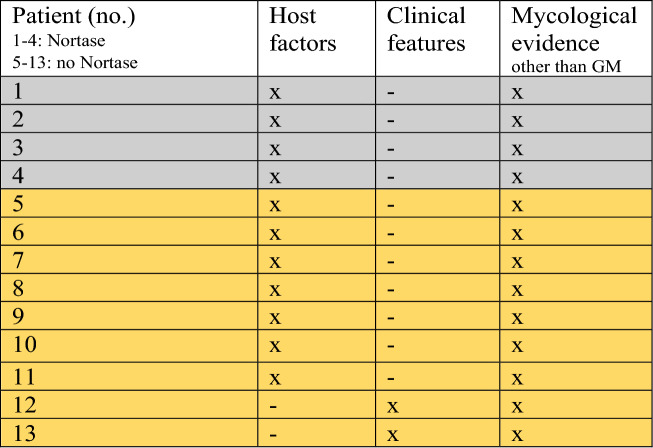
Characteristics of patients classified as presumable IA

Note: *no* number of patient, *IA* invasive aspergillosis, *grey* Nortase group, *yellow* no Nortase group

Including all patients, the positive predictive value of the GM antigen test was 0.57. When comparing the two subgroups, the test performance in group 2 with a positive predictive value (PPV) of 0.83 was significantly better than in group 1 with a positive predictive value of 0.27 (*p* < 0.001).

Chi-squared analysis demonstrated that patients allocated to group 1 had a false-positive GM test significantly more often compared to group 2 (*p* < 0.001).

### Comparison of GM levels and kinetic patterns

In all the false-positive patients in group 1, GM antigenemia occurred during the administration of Nortase. Fourteen patients (63.6%) already showed positive GM results in the first or second measurement after initiation of Nortase therapy. Five patients (22.7%) already showed negative GM results in the first or second measurement after cessation of Nortase therapy.

The median GM index in all true positive patients was 1.3, and in all false-positive patients it was 1.7. Statistical evaluation with the Mann‒Whitney *U* test showed no significant difference (*p* = 0.88) between these groups. There was also no significant difference in GM levels between true positive and false positive between each group (group 1 (*p* = 0.45), group 2 (*p* = 0.40)).

### Antifungal therapy

Systemic antifungal therapy was administered to 24 patients (80%) in group 1, and to 31 patients (88.6%) in group 2. They received voriconazole, liposomal amphotericin B, caspofungin, or isavuconazole depending on patient factors. According to our data, 16 (53.3%) patients allocated to group 1 and two patients (5.7%) allocated to group 2 retrospectively received antifungal therapy due to false-positive GM results. In contrast, none of the patients with evidence for IA was deprived of antifungal therapy in the two groups.

### Determination of GM and BDG levels in in vitro Nortase samples

In vitro analysis of Nortase samples of three different batches suspended in 10 mL normal saline each yielded a median GM index of 2.32 (range: 2.25‒7.01). The specimens were subjected to further analysis for fungal antigens by testing for the panfungal marker BDG. All Nortase batches were found to contain high concentrations of BDG (median 1034.8 pg/mL (range: 975‒1034.8)).

## Discussion

GM screening is a valuable tool for the early detection of IA [3, 4]. In immunocompromised patients, the test performance is known to be relatively good [7–9]. Our finding of a PPV of 0.83 for patients without Nortase therapy is in good agreement with the previously published data. However, we observed that 75% of our ICU patients receiving Nortase developed GM antigenemia. Deterioration of the test’s performance by the administration of digestive enzymes has not been described before. This retrospective analysis aimed to determine whether the antigen-based diagnosis was indeed correct.

The probability of IA diagnosis was graded by applying the scientific criteria for invasive mold infections defined by the EORTC/MSG consensus group [2].GM seropositivity is a major factor (criterion “mycological evidence”) for grading, but with respect to the question addressed by this study, we had to exclude antigen testing from the definitions, making them even more restrictive (modified categories: probable* and possible* IA; see Table 1). Notably, the EORTC/MSG criteria were developed for severely immunocompromised patients with cancer and for hematopoietic stem cell transplant recipients. These patients differ from typical ICU patients regarding disease presentation as well as test performances. The consensus group addressed this issue but was unable to generate recommendations for this special population [2, 11]. Alternative algorithms have been proposed for ICU patients but are not yet used in clinical routine [12]. In our study, several patients met two of the three criteria for probable IA, but not the third criterion, i.e., a clinical feature. These patients were declared as presumable IA as their specific probability for IA was high. The reason for a lack of clinical features in these patients is obvious as differences in disease presentation between neutropenic cancer patients and ICU patients are well known by now. For instance, radiological findings in ventilated patients are nonspecific so broadening the radiological criteria seems valuable [12, 13]. Therefore adding the category “presumable IA” allowed us to make the grading of ICU patients very close to the canonical EORTC/MSG guidelines (Table 1).

It is important to point out, though, that most of the IA patients (64.9%) in our study were classified along the common EORTC/MSG categories proven, probable, and possible IA (still excluding GM antigenemia).

Our data suggest that a dramatically high proportion of 73.3% of all patients receiving Nortase showed false-positive results in *Aspergillus* antigen testing (control group: 17.1%).

However, how can we tell that they were false positive?

All GM positive patients received an intense diagnostic workup, which included microbiological testing such as cultures from different body sites, CT imaging, and histopathology of the lung. Autopsy was performed in two patients, with neither histology nor culture yielding any evidence for IA. Upon exclusion of GM positivity from the IA defining criteria, these 22 and 6 patients (Nortase vs. control group) did not even meet the criteria for possible*/presumable IA; they had no evidence for infection.

GM antigenemia kinetics underlined suspected false positivity as GM levels abruptly increased within a few days after the administration of Nortase and were resolved in many cases after its discontinuation.

In vitro testing of Nortase samples of different batches yielded highly positive results in the GM and BDG assays. In a critical care setting, DEFO are usually administered through an enteral feeding tube. Presumably, impaired mucosal integrity allows for GM translocation into the bloodstream resulting in seropositivity [14]. Intestinal hyperpermeability with translocation from the gut can often be seen in critical illness [15], possibly even more relevant in critical ill patients with cystic fibrosis [16]. This might explain why the influence of DEFO on the performance of the GM test has not been described before—it might not be a problem beyond ICU settings.

In our study, more than 80% of all patients who developed GM antigenemia received systemic antifungal therapy prior to the results of a further diagnostic workup. This is concordant with a preemptive strategy, meaning antifungal therapy based on biomarker surveillance [17]. According to our data, 16 patients allocated to group 1 (53.3%), but only 2 patients allocated to group 2 (5.7%), may have retrospectively received antifungal therapy due to possibly false-positive GM results.

Our routine has been changed by these findings. The application of Nortase in patients who are at risk of IA must be strictly limited. Patients who develop GM antigenemia following DEFO application do not get antifungal therapy as long as there is no other sign of IA, as false positivity is probable. If there is any doubt, DEFO therapy should be discontinued and enteral nutrition must be initiated by peptide diets whose resorption is independent of pancreatic enzymes.

False positive GM antigenemia has been described before and was associated with the administration of generic piperacillin/tazobactam [18–22], hypercaloric drinks [23], antifungal prophylaxis [24, 25], and even ice-pop ingestion and pasta. In most cases, the proposed mechanism for false positivity was the contamination of the product with GM. Based on the literature and our in vitro results of GM and BDG testing, we assume that the applied purification techniques for digestive enzymes from fungal cultures cannot prevent contamination of the drug with cell wall polysaccharides. As in vitro testing of Nortase for BDG also turned out to be positive, this alternative antigen test cannot be expected to help confirm the diagnosis. Since BDG is a panfungal biomarker, which is also used for the diagnosis of *Candida* and *Pneumocystis* infections, we speculate that the administration of DEFO could also result in misdiagnosis of other fungal infections.

This study has certain limitations. First, the in vivo analysis has a retrospective study setting, based on a relatively low number of patients. Nevertheless, compared to previous studies that investigated potential sources of false-positive GM, this number is rather high [24, 25]. Second, the two groups differ greatly regarding age. This is linked to different underlying diseases, as DEFO are typically given to patients with end-stage cystic fibrosis following lung transplantation, who are most of the time of young age when receiving the new lung. An age- and diagnosis- dependent influence on the rate of false-positive GM tests appears not to be likely, though. Third, the case definitions of IA have been modified, resulting, in a deviation from the scientific gold standard. Therefore, a careful interpretation of the results is required, and comparability with other studies is limited.

## Conclusions

In conclusion, the GM assay allows the detection of *Aspergillus* antigen with high sensitivity and specificity in the serum of patients with IA who did not have Nortase therapy. Factors resulting in false-positive GM testing must be known to avoid misdiagnosis, unnecessary treatment, and extraneous costs. GM antigenemia in patients receiving degestive enzymes of fungal origin like Nortase should be interpreted with caution due to the significantly higher rate of false-positive test results in contrast to patients without Nortase therapy. In our opinion, the Summary of Product characteristics need to be adjusted to draw the attention of clinicians to these new findings.

## Data Availability

All data generated during this study are included in this article.

## Abbreviations

BAL: Bronchoalveolar lavage
BDG: (1,3)-Beta-D-glucan
CT: Computed tomography
DEFO: Digestive enzymes of fungal origin
ELISA: Enzyme-linked immunosorbent assay
GM: Galactomannan
IA: Invasive aspergillosis
ICU: Intensive care unit
PPV: Positive predictive value
